# Supplementary material for: SARS-CoV-2 Genome Variations in Viral Shedding of an Immunocompromised Patient with Non-Hodgkin’s Lymphoma
Source: Viruses. 2023 Jan 28;15(2):377. doi: 10.3390/v15020377 (PMC9962578; doi:10.3390/v15020377)
Supplement: Supplementary file 1 [file viruses-15-00377-s001.zip › viruses-2126138-supplementary.pdf]

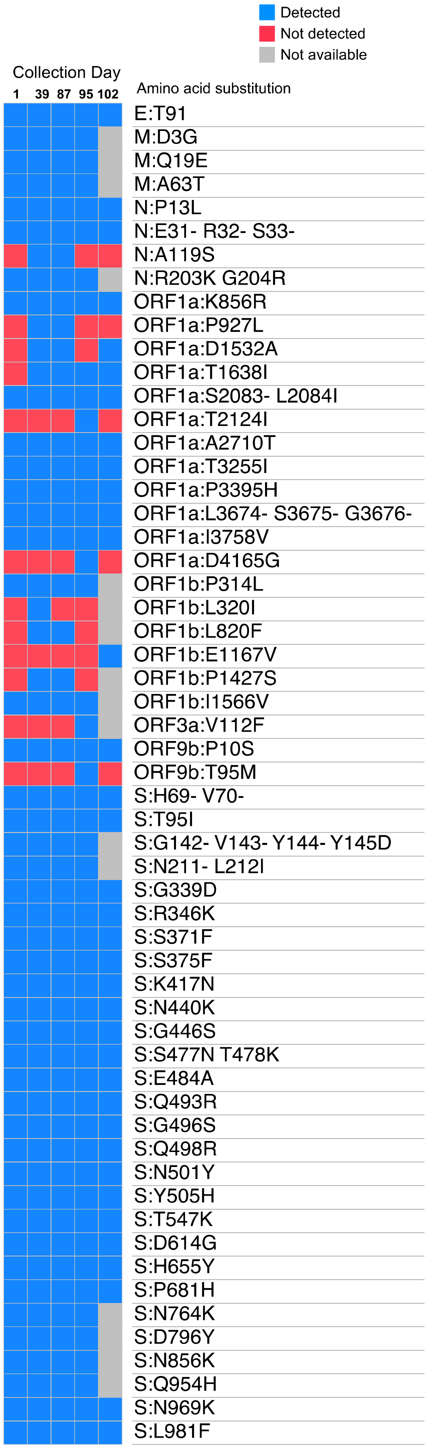

Supplementary Figure S1. Complete amino acid substitution detected during collect time. We did not find changes in the presence or absence of amino acid substitutions in the spike gene. Not available means that the region could not be covered during sequencing, so it does not have mutation data.
